# *Listeria monocytogenes* Meningoencephalitis and Cerebral Abscess in a Heart Transplant Recipient

**DOI:** 10.1155/2020/8498216

**Published:** 2020-06-21

**Authors:** Paul C. Adjei

**Affiliations:** Division of Geographic Medicine and Infectious Diseases, Tufts Medical Center, Tufts University School of Medicine Clinical and Translational Science Institute, Boston, MA, USA

## Abstract

A 54-year-old male, five months postorthotopic heart transplantation, presented with intermittent fevers, headaches, and “soupy” stools. Prior to presentation, he had low-level cytomegalovirus (CMV) viremia for two straight weeks. Given his immunosuppression, diarrhea, and low-level CMV viremia, he was presumed to have cytomegalovirus and/or *C. difficile* colitis and treated empirically for both on hospital day one. However, he developed neck pain/stiffness, diaphoresis, and worsening fevers on hospital day three. Blood cultures eventually grew *Listeria monocytogenes*; MRI of the brain with gadolinium showed left brain meningoencephalitis with early cerebral abscess formation. Lumbar puncture revealed elevated opening pressure, CSF neutrophilic pleocytosis, and elevated CSF protein and lactate but negative gram stain and cultures. First-line agent for *Listeria* meningoencephalitis is ampicillin. However, he reported amoxicillin allergy. Desensitization to ampicillin failed because ampicillin was too unstable per the allergist. He was therefore treated with penicillin monotherapy for eight weeks with complete resolution of his brain lesions and without any residual neurologic deficits.

## 1. Introduction

Listeriosis in solid organ transplant recipients is uncommon but causes high mortality. In particular, very few cases of neurolisteriosis in heart transplant recipients have been documented in the English literature. Diabetes mellitus and cytomegalovirus infection or disease are independent risk factors for this infection, whereas trimethoprim-sulfamethoxazole (TMP-SMX) prophylaxis is a protective factor. We present a case of *Listeria monocytogenes* meningoencephalitis in a heart transplant recipient who had all the above risk factors.

## 2. Case Presentation

A 54-year-old male presented with a history of end-stage ischemic cardiomyopathy for which he underwent orthotopic heart transplant. Five days after transplantation, trimethoprim-sulfamethoxazole prophylaxis against *Pneumocystis jirovecii* was replaced with atovaquone due to severe thrombocytopenia. He remained on immunosuppressive medications and valganciclovir prophylaxis as per protocol. Per protocol, his cytomegalovirus prophylaxis with valganciclovir was discontinued at three months after transplantation after which he underwent weekly laboratory testing for cytomegalovirus. Five months after transplantation, he tested positive for low-level (less than 1000 copies per milliliter) cytomegalovirus deoxyribonucleic acid (DNA) for two weeks in a row and was therefore invited to the heart infectious diseases' clinic for evaluation. In the clinic, he reported one to two weeks of malaise, fatigue, sweats, headaches, and “soupy” stools without fevers, chills, rigors, nausea, vomiting, or abdominal pain. His temperature was 39.1°C and so was admitted for further evaluation.

His past medical history included strongyloidiasis which was treated before transplantation, *Clostridioides difficile* infection, autoimmune hepatitis, and diabetes mellitus type 2.

Home medications included mycophenolate mofetil 1000 mg every 12 hours, nystatin 100,000 units swish and swallow five times per day, prednisone 15 milligrams per day, tacrolimus 5 mg in the morning and 3 mg in the evening, and glipizide 5 mg daily.

He reported that amoxicillin causes swelling and shortness of breath.

He lived at home in New Hampshire, USA, with mother and adult siblings. He originally emigrated from Laos to the USA ten years ago. He was a retired truck driver and denied pets or animal exposure, recent travels, sick contact, tobacco or alcohol use, raw or undercooked food or meat, or diary.

On physical examination, he appeared uncomfortable and mildly diaphoretic. Blood pressure was 138/82 mm Hg, heart rate 65 beats per minute, temperature 39.1°C, and respiration 18 breaths per minute on room air. Head/eyes/ear/nose/throat examination was unremarkable. Cardiopulmonary, abdominal, and neurologic examinations were normal. Skin examination was negative for rash or embolic phenomena.

On admission, results of routine blood tests were normal except for hemoglobin of 11.8 g/dL (13.5–16), sodium 133 mEq/L (135–145), creatinine 1.60 mg/dL (0.57–1.30), alanine aminotransferase 94 IU/L (0–54), aspartate aminotransferase 99 IU/L (10–52), and total bilirubin 1.7 mg/dL (0.2–1.1). Urinalysis was normal. A chest radiograph revealed mild pulmonary vascular congestion. Stool *C. difficile* test was negative. Serum CMV DNA was detected at less 1000 copies/mL.

At this stage, given his history of strongyloidiasis, immunosuppression, diarrhea, and low-level CMV viremia, our initial leading differential diagnoses were CMV colitis, *C. difficile* colitis, *Strongyloides* hyperinfection syndrome, or Gram-negative bacteria infection. *C difficile* and *Strongyloides* infections were eventually disproved with negative microbiologic and serologic testing. He was initially treated with empiric ganciclovir intravenous 2.5 milligrams/kilogram of bodyweight every 12 hours (dosed according to creatinine clearance of 53), vancomycin intravenous 1 gram every 24 hours, and meropenem intravenous 500 milligrams every 8 hours.

On the third day of admission, he developed neck pain/stiffness and worsening headaches, fevers, lethargy, and diaphoresis, raising concerns of cerebrospinal meningitis. Additional blood cultures were drawn, and he underwent lumbar puncture (LP) and magnetic resonance imaging (MRI) of the brain with gadolinium.

**Figure 1:**
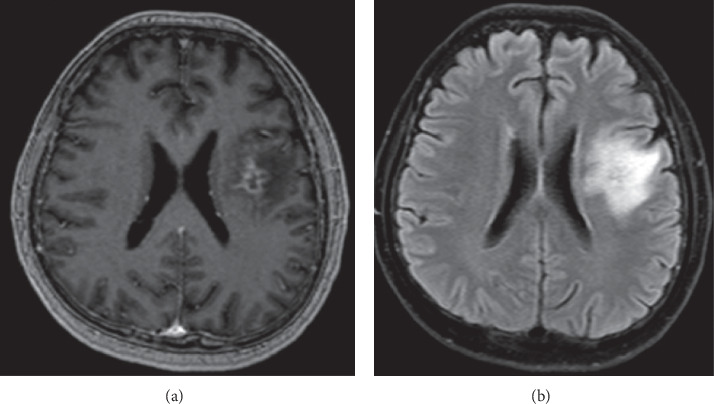
(a) Axial FLAIR v1. (b) Axial postcontrast T1 MPR. Patchy parenchymal enhancement predominantly involving the left subinsular region. The temporal operculum shows peripherally enhancing centrally cystic areas with restricted diffusion concerning for evolving meningoencephalitis with cerebral abscess formation.

All two sets of blood cultures from the day of admission eventually showed *Listeria monocytogenes* by PCR on day three (it took about four more days for growth on the culture before susceptibility testing could be performed) and changed the leading differential diagnosis to neurolisteriosis. We did not have a validated PCR test for CSF in our institution and did not have enough residual CSF to send out for testing. The patient refused additional LP as it was unlikely to change management. At this stage, ampicillin was the first choice for *Listeria*, but he had amoxicillin allergy and thus could not receive ampicillin. Therefore, pending desensitization to ampicillin, meropenem dosing was changed to central nervous system dosing of two grams every 12 hours for better central nervous system penetration. Trimethoprim-sulfamethoxazole was added for good central nervous system penetration at 320 milligrams intravenously every eight hours, and vancomycin discontinued. LP (Table 1) revealed elevated opening pressure, CSF neutrophilic pleocytosis, and elevated CSF protein and lactate but negative gram stain and cultures. MRI of the brain with gadolinium revealed left brain meningoencephalitis with early cerebral abscess formation (Figures 1(a) and 1(b)).

He underwent penicillin desensitization (ampicillin was deemed too unstable to perform desensitization), after which he was initially treated with penicillin G four million units intravenous every four hours and gentamicin (for synergistic therapeutic effect with penicillin) five milligrams/kilogram of bodyweight intravenous per day in three divided doses (peak serum gentamicin level of four to eight micrograms/milliliter and trough level of one to two micrograms/milliliter). His blood cultures from day three of admission remained negative. His central nervous system symptoms resolved approximately six days into penicillin/gentamicin therapy. Gentamicin was discontinued on day six of therapy because his gross central nervous system symptoms had resolved and because he was post-heart transplantation patient with high risk of acute kidney injury who was already on potentially nephrotoxic immunosuppressive medications.

**Figure 2:**
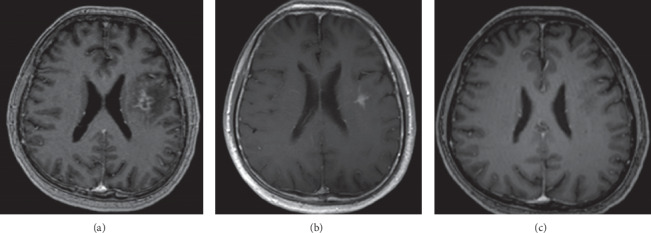
Axial FLAIR MRI images on admission, week 4 of therapy, and week 8 of therapy, respectively, showing resolution on treatment: (a) week 0, (b) week 4, and (c) week 8.

He was discharged eleven days after admission. He received a total of nine weeks of penicillin G monotherapy as guided by outpatient brain MRI. His outpatient brain imaging studies showing resolution are shown in Figures 2(a)–2(c). He did not suffer any residual neurologic deficits. This shows that penicillin monotherapy is likely as good as ampicillin for neurolisteriosis.

## 3. Discussion

CNS infections are among the rarer infections seen in transplant patients and are usually caused by opportunistic pathogens such as herpes viruses, filamentous fungi, *Cryptococcus neoformans*, and *Toxoplasma gondii*, but bacteria are also causative organisms [1–3]. Listeriosis in solid organ transplant recipients is uncommon but causes high mortality. In particular, very few cases of neurolisteriosis in heart transplant recipients have been documented in the English literature. We found only seven reported cases in six patients from 1989 [4, 5]. Diabetes mellitus and cytomegalovirus infection or disease are independent risk factors for this infection, whereas TMP-SMZ prophylaxis is a protective factor [4, 6]. Because of its immunomodulatory effects and associated neutropenia, cytomegalovirus (CMV) infection/disease in transplant recipients predisposes to a variety of devastating bacterial and fungal infections including listeriosis [4]. Thus, CMV prophylaxis in such patients might prevent not only CMV infection/disease but also other infections such as listeriosis [4, 6]. In a series of five patients (out of a total of 3500 heart transplant recipients observed over a 27-year period), three of the four patients who died developed CMV disease before developing listeriosis; meanwhile, none of the patients was on trimethoprim-sulfamethoxazole prophylaxis for *Pneumocystis jirovecii* (PJP) [4, 6]. PJP prophylaxis specifically with trimethoprim-sulfamethoxazole has been found to be protective against *Listeria monocytogenes* and other organisms, including *Nocardia* spp., *Toxoplasma gondii*, and *Legionella* spp [4, 6, 7]. Our patient had had all the above risk factors [4, 6].

Work-up for suspected *Listeria* meningoencephalitis includes blood cultures, lumbar puncture, and MRI of the brain. In the setting of fever and high clinical suspicion for meningoencephalitis, empiric treatment with broad spectrum antibiotics including ampicillin (or penicillin) should be administered. Diagnosis is based upon cerebrospinal fluid and blood cultures growing *Listeria monocytogenes*. Blood cultures tend to be of higher yield (approximately 70%) than cerebrospinal fluid (CSF) cultures (approximately 40% in meningoencephalitis and anywhere from zero to twenty percent in abscess/cerebritis) [8, 9]. Analysis of the cerebrospinal fluid (CSF) shows pleocytosis, ranging from 100% polymorphonuclear cells to 100% mononuclear cells. It is the one cause of bacterial (nontuberculous) meningitis in which lymphocytic pleocytosis can be seen in the CSF in the absence of antibiotic therapy (our patient was on *Listeria*-effective antibiotics and had low CSF lymphocytes) as well as elevated CSF lactate (which our patient had). CSF protein concentration is usually moderately elevated, and low glucose concentrations were found in about one third of patients. The diagnostic imaging of choice is brain MRI as CT is often normal early in the disease process [8, 9]. Given the difficulties, isolating the organism with conventional cultures and the bad outcomes of untreated or inappropriate antibiotic treatment, a more rapid and accurate diagnostic method is required. Real-time PCR (RT-PCR) has been shown to be a highly promising diagnostic modality in identifying the presence of the organism in the CSF. The highly conserved and specific hyl gene proved to be a reliable replication target with no false-negative results shown, and the turnaround time for RT-PCR is 2 hours [10]. For instance, the early availability of serum PCR results in our patient (four full days ahead of culture and sensitivity results) informed crucial treatment decisions and was potentially lifesaving. Ampicillin is the first choice for *Listeria*, but penicillin is an equally effective treatment as in our patient.

Finally, even though CSF Gram stain and culture were negative in our patient, the complete resolution of MRI brain findings after nine weeks of therapy was proof that the diagnosis was correct.

## Figures and Tables

**Table 1:** Cerebrospinal fluid analysis result.

Color	Tube 1	Tube 4
	Slightly cloudy	Slightly cloudy
Nucleated cells (0–5)/*μ*L	615	435
Neutrophils (0–6), %	58	48
Lymphocytes (40–80), %	7	7
Monocytes/macrophages (15–45), %	34	45
Red blood cell count < 1/*μ*L	24	9
Protein (15–45), mg/dL	172	Not done
Glucose (38–45), mg/dL	72 (38–85)	Not done
CSF lactate (0.5–2.0), mEq/L	6.9 (0–2)	Not done
Opening pressure, cm of water	39	Not done
Gram stain and culture	Negative	Not done
